# Supplementary material for: Bioaccessibility of Bioactive Compounds of ‘Fresh Garlic’ and ‘Black Garlic’ through In Vitro Gastrointestinal Digestion
Source: Foods. 2020 Oct 31;9(11):1582. doi: 10.3390/foods9111582 (PMC7693347; doi:10.3390/foods9111582)
Supplement: Supplementary file 1 [file foods-09-01582-s001.pdf]

SUPPLEMENTARY INFORMATION

Bioaccessibility of Bioactive Compounds of ‘Fresh Garlic’ and ‘Black Garlic’ Through *In-Vitro* Gastrointestinal Digestion.

Alicia Moreno-Ortega[a,b], Gema Pereira-Caro[a*], José Luis Ordóñez[a], Rafael Moreno-Rojas[b], Víctor Ortíz-Somovilla[a], José Manuel Moreno-Rojas[a*]

[a]Department of Food Science and Health, Andalusian Institute of Agricultural and Fisheries Research and Training (IFAPA), Alameda del Obispo, Avda. Menéndez-Pidal, s/n. 14004, Córdoba, Spain

[b]Departamento de Bromatología y Tecnología de los Alimentos, Campus Rabanales, Ed. Darwin-anexo Universidad de Córdoba, 14071, Córdoba, Spain

*Corresponding authors:
Gema Pereira-Caro (mariag.pereira@juntadeandalucia.es)
José Manuel Moreno-Rojas (josem.moreno.rojas@juntadeandalucia.es)
IFAPA-Alameda del Obispo, Department of Food Science and Health, Avenida Menéndez Pidal, S/N, 14071 Córdoba (Spain). Tel. 0034 671 532 734

Table S1. Details of the Simulated Salivary Fluid (SSF), Simulated Gastric Fluid (SGF) and Simulated Intestinal Fluid (SIF)

	**$MgCl_2(H_2O)_6$ (0.15 M)**	**KCl (0.5 M)**	**KH_2PO_4 (0.5 M)**	**$(NH_4)_2CO_3$ (0.5 M)**	**$NaHCO_3$ (1 M)**	**NaCl (2 M)**	**Distilled Water**	**Final Volume**
				**mL**				
**SSF**	0.313	9.438	2.313	0.038	4.250	-	233.650	**250.0**
**SGF**	0.250	4.313	0.563	0.313	7.813	7.375	229.375	**250.0**
**SIF**	0.688	4.250	0.500	0.688	26.563	6.000	211.313	**250.0**

Table S2. HPLC-HRMS-Based Identifications of (Poly)phenols in Fresh and Black Garlics

Peak	RT	Compound	Chemical Formula $[m/z]^-$	Experimental Mass $[m/z]^-$	δ (ppm)	MSI MI level[a]
**1**	4.7	Epigallocatechin	C15H13O7	305.066	-0.48	2
**2**	7.2	Benzoic Acid	C7H5O2	121.028	0.68	1
**3**	8.0	Chlorogenic acid	C16H17O9	353.087	-1.13	1
**4**	8.2	Catechin	C15H13O6	289.071	-1.09	1
**5**	8.3	Caffeic acid	C9H7O4	179.034	0.28	1
**6**	9.7	Gallic acid	C7H5O5	169.013	-2.15	1
**7**	11.7	Epicatechin	C15H13O6	289.071	-1.09	1
**8**	18.9	Vanillic acid	C8H7O4	167.034	0.67	2
**9**	21.1	Ferulic acid	C10H9O4	193.050	1.79	1
**10**	24.4	*p*-Coumaric acid	C9H7O3	163.039	-1.96	1

[a]Metabolite standards initiative (MSI) metabolite identification (MI) levels [1]. Reference compounds were available for all compounds identified at MSI MI level 1

Table S3. HPLC-HRMS-Based Identifications of organosulfur compounds in Fresh and Black Garlics

Peak	RT	Compound	Chemical Formula [m/z]+	Experimental Mass [m/z]+	δ (ppm)	MSI MI level[a]
**1**	4.2	S-Allylmercapto-L-cysteine (SAMC)	C6H12NO2S2	194.030	0.85	2
**2**	4.6	S-Allylcysteine (SAC)	C6H12NO2S	162.058	-1.9	1
**3**	5.7	S-Methylcysteine (Deoxymethiin)	C4H10NO2S	136.043	0.11	2
**4**	5.8	S-Propyl-L-cysteine sulfoxide (Propiin)	C6H14NO3S	180.069	2.45	2
**5**	5.9	S-(S-propyl) cysteine	C6H14NO2S2	196.046	0.36	2
**6**	5.9	Cycloalliin	C6H12NO3S	178.053	-1.36	2
**7**	6.6	γ –Glutamyl-S-(S-1-propenyl) cysteine (GS1PC)	C11H19N2O5S	291.101	1.65	2
**8**	6.7	γ-Glutamyl-S-allylmercaptocysteine (GSAMC)	C11H19N2O5S2	323.073	-1.9	2
**9**	6.8	S-Allylsulfenic acid (Lacrimatory factor)	C3H7OS	91.021	3.42	2
**10**	6.8	N-Acetyl-S-allyl-L-cysteine (NASAC)	C8H14NO3S	204.069	-1.39	2
**11**	6.8	Alliin	C6H12NO3S	178.053	-2.82	1
**12**	6.8	γ –Glutamyl-S-(S-1-propenyl)cysteine-glycine	C13H22N3O6S2	380.094	0.92	2
**13**	7.0	S-(2-carboxypropyl) cysteine	C7H14NO4S	208.064	-2.15	2
**14**	7.1	Trans-S-(1-propenyl)-L-cysteine (S1PC)	C6H12NO2S	162.058	-0.70	2
**15**	7.3	γ −Glutamyl-S-allyl-L-cysteine (GSAC)	C11H19N2O5S	291.101	1.86	2
**16**	7.4	S-Allylglutathione	C13H22N3O6S	348.122	-0.23	2
**17**	7.6	S-Methyl-l-cysteine sulfoxide (Methiin)	C4H10NO3S	152.038	-1.33	2
**18**	7.6	γ –Glutamyl-S-(S-methyl)cysteine-glycine	C11H20N3O6S2	354.079	-0.51	2
**19**	7.7	Methionine sulfoxide	C5H11NO3S	166.053	1.94	2
**20**	8.5	γ -Glutamyl-cysteine	C8H15N2O5S	251.070	-2.45	2
**21**	8.6	γ –Glutamyl-S-(2-carboxypropyl)cysteine-glycine	C14H24N3O8S	394.128	0.67	2
**22**	9.0	γ-Glutamyl-S-(1-propenyl) cysteine sulfoxide (G1PCS)	C11H19N2O6S	307.096	-2.05	2
**23**	9.2	ɣ-Glutamyl-S-methyl-L-cysteine (GSMC)	C9H17N2O5S	265.085	1.56	2
**24**	10.0	ɣ −Glutamyl-S-methyl-L-cysteine sulfoxide (GSMCS)	C9H17N2O6S	281.080	2.19	2

[a]Metabolite standards initiative (MSI) metabolite identification (MI) levels [1]. Reference compounds were available for all compounds identified at MSI MI level 1

References

1. Sumner, L.W.; Amberg, A.; Barrett, D.; Beale, M.H.; Beger, R.; Daykin, C.A.; W-M Fan, T.; Fiehn, O.; Goodacre, R.; Griffin, J.L.; et al.

Proposed minimum reporting standards for chemical analysis Chemical Analysis Working Group (CAWG) Metabolomics Standards Initiative (MSI) *NIH Public Access. Metabolomics* **2007**, *3*, 211–221, doi:10.1007/s11306-007-0082-2.
